# Motivation for whole-heart perfusion CMR: a simulation study based on retrospective comparison of the diagnostic performance of 3-slice vs. whole-heart SPECT

**DOI:** 10.1186/1532-429X-16-S1-O99

**Published:** 2014-01-16

**Authors:** Behzad Sharif, Reza Arsanjani, C Noel Bairey Merz, Daniel S Berman, Debiao Li, Piotr Slomka

**Affiliations:** 1 Biomedical Imaging Research Institute, Cedars-Sinai Medical Center, Los Angeles, California, USA; 2 Medicine, Cedars-Sinai Medical Center, Los Angeles, California, USA

## Background

There is an ongoing debate on the merit of whole-heart 3D CMR perfusion imaging in terms of improving the overall diagnostic accuracy of myocardial perfusion CMR [1]. Whole-heart imaging is technically more challenging [2] and requires a compromise in terms of the spatial resolution [2] and temporal footprint (hence, potentially leading to more instances of artifactual images). The purpose of this work was to evaluate the potential incremental diagnostic value of whole-heart myocardial perfusion imaging compared to 3-slice imaging. The presented results are based on on a simulation of 3-slice imaging with myocardial perfusion SPECT (MPS) in a retrospective quantitative analysis of a large cohort who underwent stress MPS [3,4].

## Methods

A total of n = 995 patients (n = 504 males) with suspected CAD underwent Tc-99-Sestamibi rest/stress MPS, n = 650 of which had correlative angiography within 60 days and the remainder n = 345 patients where considered low likelihood (< 5% based on Diamond and Forrester criteria) [3,4]. Stress total perfusion deficit (TPD) [4] was derived using the Quantitative Perfusion SPECT (QPS) software [5] from MPS images. The "whole-heart TPD" for a given patient was computed by integrating the abnormal stress hypoperfusion severities obtained from the maximum count profiles normal to the left ventricular surface and using an mean absolute deviation threshold of 3.0 as compared to normal perfusion (based on low-likelihood patient database) [4,5]. This approach is a current clinical standard for MPS and is equivalent to expert visual read [6]. The "3-slice TPD" was computed by limiting the MPS data to a subset of only 3 short-axis slices (each approximately 10 mm) at apical, mid and basal position as practiced in CMR, by subsampling the whole-heart MPS data. The diagnostic performance of whole-heart vs. 3-slice TPD was compared using receiver operating curves (ROCs). Furthermore, correlation between the two methods were compared using linear regression.

## Results

**Figure 1:**
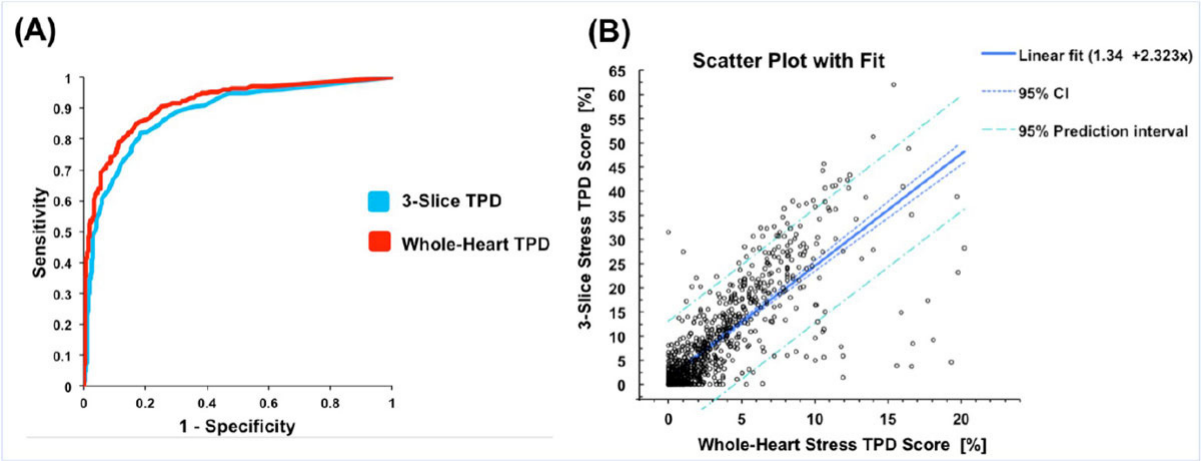
**(A) Receiver operating curves (ROCs) for whole-heart TPD and 3-slice TPD using invasive coronary angiography as the gold standard (≥70% stenosis considered significant)**. The area under the curve (AUC) for diagnostic performance based on 3-slice TPD is significantly lower compared to whole-heart TPD (AUC for 3-slice = 0.88 ± 0.01, AUC for whole-heart = 0.91 ± 0.01, p < 0.0001). (B) Scatter plot showing regression of 3-slice TPD against whole-heart TPD demonstrating a moderate correlation (R^2 = 0.65, p < 0.0001).

Figure 1A shows the ROCs for detection of significant CAD for whole-heart TPD and 3-slice TPD with invasive coronary angiography as the gold standard. The area under the curve (AUC) for 3-slice TPD is significantly lower compared to whole-heart TPD (0.88 ± 0.01 vs. 0.91 ± 0.01, p < 0.0001). Figure 1B shows a scatter-plot and linear regression of 3-slice TPD against whole-heart TPD demonstrating a moderate correlation (R2 = 0.65, p < 0.0001).

## Conclusions

The diagnostic performance of whole-heart TPD is significantly superior to the 3-slice model of TPD with moderate correlation between the TPD scores. Despite technical differences between the CMR and MPS modalities, these results show added value of whole heart perfusion imaging and provide motivation for pursuing 3D whole-heart perfusion CMR techniques.

## Funding

Grant sponsors: National Institutes of Health grants nos. NHLBI R01HL089765, R01HL38698. American Heart Association Postdoctoral Fellowship Award 11POST7390063.
